# Recurrent meningioma treated with boron neutron capture therapy: a feasibility study with dosimetric and clinical correlates

**DOI:** 10.1007/s11060-025-05184-w

**Published:** 2025-07-31

**Authors:** Tien-Li Lan, Chun-Fu Lin, Yi-Yen Lee, Feng-Chi Chang, Shih-Chieh Lin, Fong-In Chou, Jinn-Jer Peir, Po-Shen Pan, Jen-Kun Chen, Lu-Han Lai, Hiroki Tanaka, Shih-Ming Hsu, Yi-Wei Chen

**Affiliations:** 1 School of Medicine, National Yang Ming Chiao Tung University, Taipei, Taiwan; 2 Department of Biomedical Imaging and Radiological Sciences, National Yang Ming Chiao Tung University, Taipei, Taiwan; 3 Department of Neurosurgery, Neurological Institute, Taipei Veterans General Hospital, Taipei, Taiwan; 4 Department of Radiology, Taipei Veterans General Hospital, Taipei, Taiwan; 5 Department of Pathology and Laboratory Medicine, Taipei Veterans General Hospital, Taipei, Taiwan; 6 Nuclear Science & Technology Development Department, National Tsing-Hua University, Hsinchu City, Taiwan; 7 Department of Chemistry, Tamkang University, New Taipei City, Taiwan; 8 Institute of Biomedical Engineering and Nanomedicine, National Health Research Institute, Zhunan Town, Miaoli County, Taiwan; 9 Department of Medical Imaging and Radiological Technology, Yuanpei University of Medical Technology, Hsinchu, Taiwan; 10 Particle Radiation Oncology Research Center, Institute for Integrated Radiation and Nuclear Science, Kyoto University, Osaka, Japan; 11 Department of Heavy Particles and Radiation Oncology, Taipei Veterans General Hospital, Taipei, Taiwan

**Keywords:** Boron neutron capture therapy, Benign meningioma, Atypical meningioma, Fluciclovine

## Abstract

**Background:**

Boron neutron capture therapy (BNCT) is a targeted radiotherapy modality that has shown promise in the treatment of recurrent gliomas and head and neck cancers. Although meningiomas are generally slow-growing, recent studies have demonstrated favorable uptake of boron-containing compounds, particularly boronophenylalanine (BPA), suggesting a potential role for BNCT in recurrent meningioma.

**Methods:**

We retrospectively analyzed 13 patients with recurrent meningiomas treated with salvage BNCT at the Tsing Hua Open-Pool Reactor between August 2020 and May 2024. Tumor uptake was assessed using either ¹⁸F-BPA or ¹⁸F-Fluciclovine PET. Treatment response was evaluated using RANO criteria, and outcomes were analyzed in relation to dosimetric and clinical factors.

**Results:**

Of the 13 patients (1 WHO grade 3, 6 grade 2, and 6 grade 1), 5 (38%) responded to BNCT. Responders had significantly higher tumor mean dose (45.10 vs. 25.85 GyE, *p* = 0.003). Tumor location influenced dosimetry; non–skull base tumors received higher doses and showed a trend toward better response. TNR and tumor size were not predictive of response. No severe adverse events were observed.

**Conclusions:**

Salvage BNCT is a feasible and well-tolerated treatment for recurrent meningioma, with dose distribution and tumor location significantly influencing treatment response. Further studies are warranted to refine imaging and planning strategies, particularly for skull base lesions and in the context of Fluciclovine PET.

## Introduction

Meningiomas are the most common type of intracranial tumor, accounting for over 30% of all primary brain and central nervous system tumors diagnosed in adults [1]. While the majority of meningiomas are benign and exhibit slow growth, certain subtypes display more aggressive behavior and are classified as atypical or anaplastic meningiomas [2]. Common symptoms include increased intracranial pressure and focal neurological deficits, such as limb weakness or visual disturbances, depending on the tumor’s location. Given the typically long life expectancy of affected patients [3], treatment strategies must carefully balance the goal of effective tumor control with the potential for treatment-related neurological impairment.

Surgical resection via craniotomy remains the primary treatment for symptomatic or aggressive meningiomas [4]. However, several anatomical and pathological factors complicate complete surgical removal. Previous studies have shown that skull base meningiomas are more frequently low-grade; yet, their proximity to critical neurovascular structures and high vascularity often make gross total resection (Simpson Grade I–II) difficult to achieve. Conversely, meningiomas located on the convexity are more likely to be higher-grade (WHO Grade 2 or 3) and are generally more accessible surgically [5, 6]. Nonetheless, their tendency to invade adjacent brain parenchyma can hinder the achievement of complete resection [7].

For WHO Grade 3 meningiomas (also referred to as anaplastic or malignant meningiomas), adjuvant radiotherapy is universally recommended following surgery. In the case of WHO Grade 2 meningiomas (atypical meningiomas), adjuvant radiotherapy is typically reserved for patients who have undergone subtotal resection [8].

Management of recurrent meningiomas presents additional challenges. Prior radiotherapy may preclude re-irradiation due to dose constraints affecting nearby normal tissues, particularly when recurrence occurs shortly after initial treatment [9]. Advanced radiation techniques with superior dose distribution properties have been investigated for re-irradiation in such cases, including Gamma Knife radiosurgery [10], proton therapy [11], and carbon ion radiotherapy [12]. Currently, no systemic therapies have received FDA approval specifically for meningioma. Some studies have suggested that hydroxyurea and sunitinib may offer limited benefit in recurrent cases [13, 14], while bevacizumab has been used to alleviate peritumoral edema [15]. However, further clinical trials are needed to establish the efficacy of these agents.

Boron neutron capture therapy (BNCT) has emerged as a promising therapeutic approach, offering selective tumor cell destruction with minimal impact on surrounding healthy tissue [16, 17]. BNCT relies on the selective uptake of boron-10-containing compounds by tumor cells. Upon neutron irradiation, boron-10 undergoes a nuclear reaction that produces an alpha particle and a lithium nucleus, with path lengths of approximately 9 μm and 4 μm, respectively. This short-range, high-linear energy transfer radiation allows for precise tumor cell targeting while sparing normal tissue [18].

The efficacy of BNCT depends primarily on two factors: the differential uptake of boronated compounds between tumor and normal tissue, and the availability of an appropriate epithermal neutron source [19, 20]. In the context of meningiomas, boronophenylalanine (BPA)—a boron-containing amino acid derivative—has demonstrated favorable tumor-to-normal tissue uptake ratios and has shown promising therapeutic outcomes in several preclinical and clinical studies [21, 22].

In this study, we present the clinical outcomes of patients with recurrent meningioma treated with salvage BNCT at the Tsing-Hua Open-Pool Reactor (THOR) since 2020. We also analyze key factors influencing treatment response and tumor control.

## Methods

### Study design and patient selection

This study is a retrospective analysis of patients diagnosed with meningioma who received salvage BNCT following tumor recurrence after initial treatment. Meningiomas were classified as benign, atypical, or anaplastic based on pathological findings and the WHO grading system [23]. Patient characteristics are summarized in Table 1.

**Table 1:** Baseline characteristics of the 13 patients with recurrent meningioma treated with salvage BNCT

Variable	*n* = 13 (%)	Mean (SD)	Median (Range)
**Gender**			
Male	5 (38)		
Female	8 (62)		
**Age**		62.84 (11.70)	68 (42–82)
**F-BPA TNR**	4	2.48 (0.57)	2.47 (1.97–3.02)
**Fluciclovine TNR**	9	8.83 (3.49)	8.87 (2.66–14.14)
**Tumor site**			
Non-skull base	9 (69)		
Skull base	4 (31)		
**Tumor size (cc)**		55.30 (31.67)	59.59 (6.67-118.55)

BNCT was administered between August 2020 and May 2024, with a total of 17 treatment courses performed during this period. This study was approved by the Institutional Review Board of Taipei Veterans General Hospital (IRB-TPEVGH No. 2024-06-005 A, Protocol No. TBCGHPZ20231218). Informed consent was obtained from all individual participants included in the study. After excluding patients who underwent a second BNCT course or had less than six months of post-treatment follow-up, 13 patients were included in the final analysis.

All patients underwent pre-BNCT PET imaging to assess treatment eligibility. Prior to June 2022, ¹⁸F-BPA PET was used as the radiotracer; after June 2022, ¹⁸F-Fluciclovine PET was adopted. A minimum tumor-to-normal tissue ratio (TNR) of 2.0—defined as the ratio of mean standardized uptake values (SUV) between the tumor and the contralateral cerebellum—was required for treatment eligibility.

### BNCT treatment protocol

All BNCT treatments were delivered at the THOR, operating at an epithermal neutron flux of 1.69 × 10⁹ n/cm²·s and a power output of 2 MW. Neutron beam quality and contamination were maintained according to the International Atomic Energy Agency (IAEA) guidelines (2023) [24].

For patients evaluated with ¹⁸F-BPA PET, the specific TNR value was incorporated into the treatment planning system for tumor dose calculation. In contrast, for patients evaluated with ¹⁸F-Fluciclovine PET—given that previous studies have shown Fluciclovine does not reliably correlate with BPA uptake in tumor tissue—a fixed TNR value of 2.5 was used for dose planning [25]. This constant was based on the average TNR observed in prior ¹⁸F-BPA PET studies of meningioma [26].

Irradiation time and dose were determined by either the constraint of a maximum brain dose of 13 Gy-equivalent (GyE) or a mean brain dose of 3 GyE, whichever threshold was reached first. No specific dose constraint was applied to the tumor.

### Imaging and response evaluation

Magnetic resonance imaging (MRI) was performed before BNCT and again three months after treatment to assess therapeutic response. Tumor response was evaluated according to the Response Assessment in Neuro-Oncology (RANO) criteria [27]. Patients achieving complete response (CR) or partial response (PR) were categorized as responders, while those with stable disease (SD) or progressive disease (PD) were considered non-responders.

### Survival and statistical analysis

All patients were followed for a minimum of six months. The primary endpoint was the tumor response rate following BNCT. Progression-free survival (PFS) was defined as the interval from BNCT to either radiographic or clinical disease progression, or the last follow-up visit. PFS was analyzed using Kaplan–Meier survival curves and compared using the log-rank test. Independent t-tests were conducted to compare treatment responses between groups. All statistical analyses were performed using SPSS software.

## Results

A total of 13 patients were included in this study, comprising 5 males and 8 females. Among them, 1 patient was diagnosed with WHO grade 3 anaplastic meningioma, 6 with WHO grade 2 atypical meningioma, and 6 with WHO grade 1 benign meningioma. All cases represented recurrences following prior external beam photon radiotherapy (dose range: 30–40 Gy). Most patients had undergone craniotomy as part of their initial treatment. However, 2 patients with skull base meningioma were unable to undergo surgery due to the complexity of the operative approach, and 1 patient with parasagittal meningioma declined surgical intervention.

The mean age of the cohort was 62.85 years (range: 42–82 years). The mean TNR for patients evaluated with F-BPA PET was 2.48 ± 0.57 (range: 1.97–3.02), while for those evaluated with Fluciclovine PET, the mean TNR was 8.83 ± 3.49 (range: 2.66–14.14). The mean tumor volume was 55.30 ± 31.67 cm³, with the largest tumor measuring 118.55 cm³ (Table 1). The median follow-up duration for this cohort was 14.55 months.

**Fig. 1:**
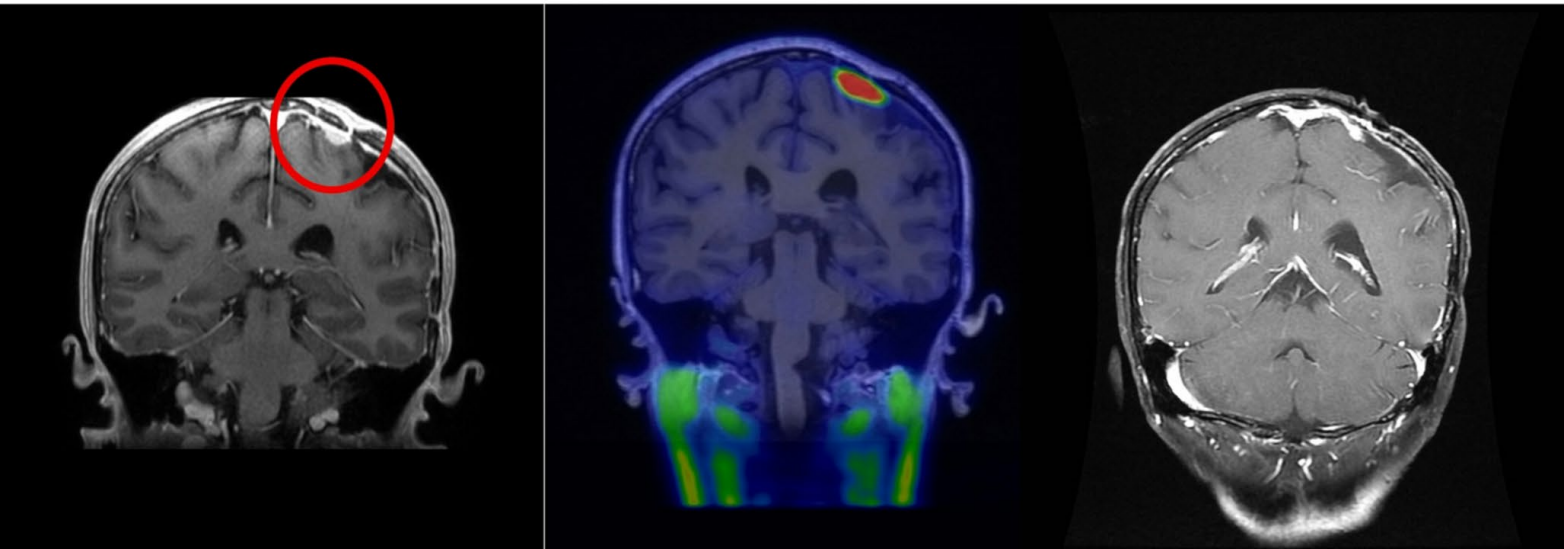
Representative case of a patient with recurrent convexity atypical meningioma. (Left) Coronal MRI image prior to BNCT, with the tumor delineated by a red circle. (Middle) Pre-treatment Fluciclovine PET scan showing high radiotracer uptake; the tumor-to-blood SUV ratio was 11.58, indicating high expression of amino acid transporters. (Right) Coronal MRI image two months after BNCT showing marked tumor shrinkage, consistent with a complete response

All patients received a single session of BNCT. The average irradiation time was 1,484 ± 458 s (range: 734–2,369 s), determined by normal tissue dose constraints. Two patients reached the brain maximum dose constraint of 13 GyE, while the remainder reached the brain mean dose constraint of 3 GyE. The mean tumor dose was 33.25 ± 13.77 GyE (range: 14.91–55.04 GyE), and the minimum tumor dose was 19.16 ± 10.71 GyE (range: 5.28–42.56 GyE) (Fig. 1).

### Tumor location and dose distribution

**Fig. 2:**
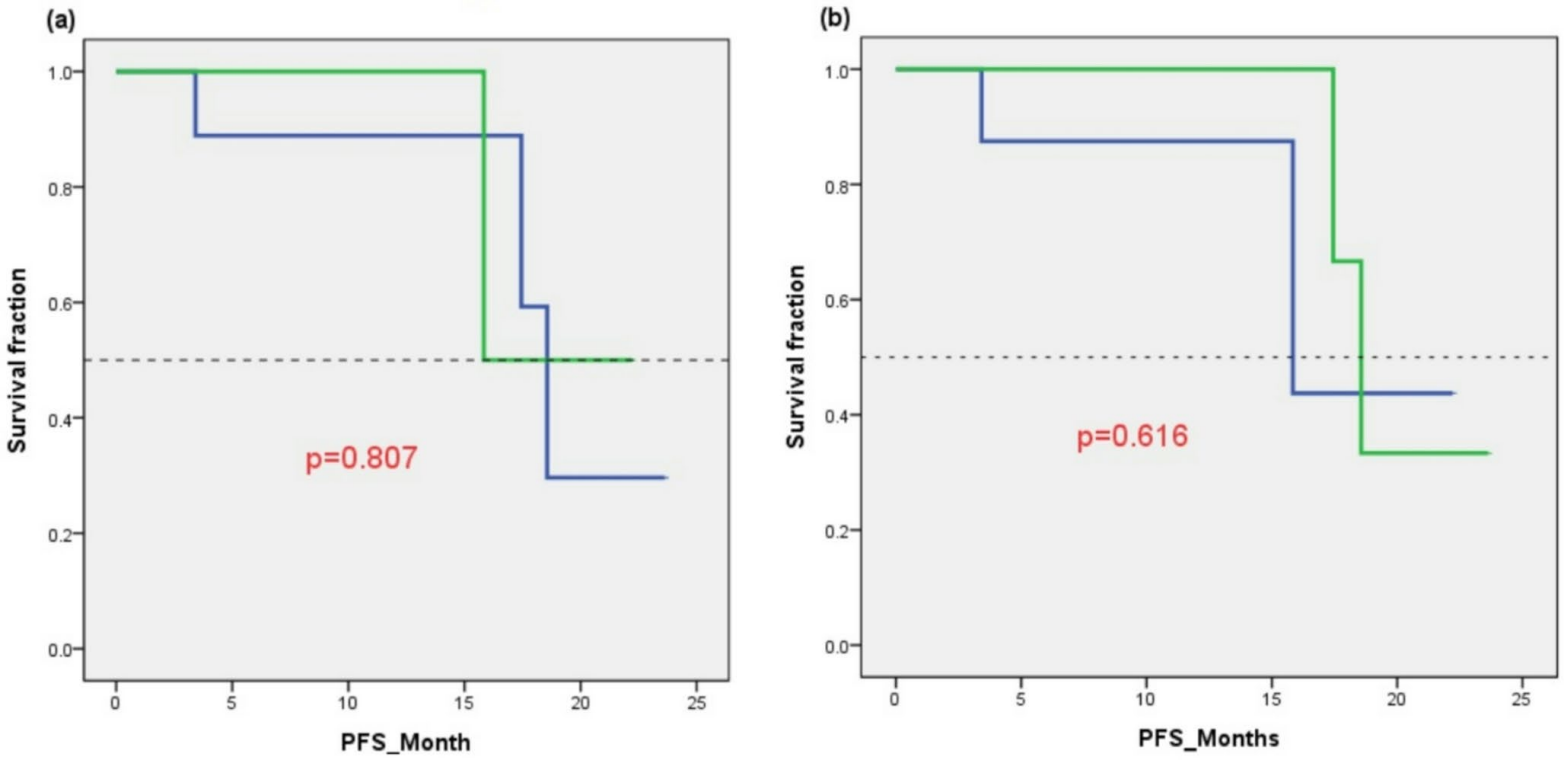
(**a**) Kaplan–Meier survival curve for progression-free survival (PFS) stratified by tumor location. The green line represents skull base meningiomas; the blue line represents non–skull base meningiomas. (**b**) Kaplan–Meier survival curve for PFS stratified by treatment response. The green line represents responders (complete or partial response); the blue line represents non-responders (stable or progressive disease)

We first compared treatment outcomes based on tumor location, categorizing 9 patients with non–skull base meningiomas and 4 with skull base meningiomas. There was no significant difference in WHO tumor grade distribution between the two groups (Fisher’s exact test, *p* = 0.27). Tumor volume also did not differ significantly (52.02 vs. 62.68 cm³, *p* = 0.598). However, significant differences were observed in dosimetric parameters: mean tumor dose was higher in non–skull base tumors (40.77 vs. 16.33 GyE, *p* < 0.001), as was minimum tumor dose (23.04 vs. 10.44 GyE, *p* = 0.04). These differences are likely attributable to the greater penetration depth of the epithermal neutron beam in non–skull base locations. This may have contributed to a trend toward a higher response rate in non–skull base meningiomas, with 5 patients (56%) responding to BNCT, compared to none in the skull base group (*p* = 0.10). However, this did not translate into a PFS benefit (median PFS: 18.56 vs. 15.84 months, *p* = 0.807) (Fig. 2a).

### Predictors of treatment response

We next investigated factors associated with response to BNCT. Patients categorized as responders (complete or partial response) received significantly higher mean tumor doses compared to non-responders (45.10 vs. 25.85 GyE, *p* = 0.003). Responders also tended to receive higher minimum tumor doses (27.39 vs. 14.02 GyE, *p* = 0.055). Tumor volume (39.81 vs. 64.98 cm³, *p* = 0.17) and TNR (5.63 vs. 7.66, *p* = 0.42) were not significantly associated with treatment response. Additionally, response status did not significantly affect PFS (median PFS: 18.56 vs. 15.84 months, *p* = 0.616, log-rank test) (Fig. 2b). Tumor grade also had no significant impact on PFS; patients with WHO grade 1 meningioma had a similar PFS compared to those with grade 2 or 3 tumors (median PFS: not reached vs. 18.56 months, *p* = 0.568).

### Treatment-related adverse events

Adverse events were minimal. One patient experienced a single seizure episode one month after BNCT, which resolved spontaneously without medication and did not recur. Another patient experienced grade 1 alopecia. No severe (grade ≥ 2) adverse events were reported in this cohort.

## Discussion

Boron neutron capture therapy has gained increasing attention in recent years, with over 400 treatment cases performed at the Tsing Hua Open-Pool Reactor. Initially developed for the treatment of aggressive malignancies such as recurrent glioblastoma, head and neck cancers, and melanoma [28, 29], BNCT has also demonstrated unexpected potential in treating recurrent meningiomas [26, 30, 31]. Despite their typically indolent behavior, meningiomas have shown high uptake of boron-containing compounds, particularly BPA, supporting the feasibility of BNCT as a salvage therapy in this population [32, 33].

The extension of BNCT to meningiomas requires strict adherence to pre-treatment eligibility criteria, particularly confirmation of adequate tumor-to-normal tissue uptake, quantified as the tumor-to-normal tissue ratio. Historically, F-BPA PET—produced by labeling BPA directly with fluorine-18—has served as the gold standard for BNCT planning [34]. However, due to restrictions on the availability of F-BPA since the end of 2022, alternative tracers have been explored. Fluciclovine, initially developed for detecting prostate cancer metastases [35], shares the same transporter (L-type amino acid transporter 1, LAT1) with BPA [36]. However, Fluciclovine can also enter cells via the ASCT2 transporter, resulting in significantly higher tumor uptake than BPA [37, 38].

In our study, patients undergoing F-BPA PET demonstrated a mean TNR of approximately 2.5, whereas those evaluated with Fluciclovine PET had a markedly higher mean TNR of 8.8. This discrepancy leads to an overestimation of BPA uptake when Fluciclovine PET is used, limiting the precision of dose calculations in treatment planning. As a solution, we applied a standardized TNR value of 2.5 for dose calculation in patients evaluated with Fluciclovine PET, based on the average TNR observed in prior F-BPA PET studies of meningioma.

Previous BNCT studies in recurrent gliomas have shown that TNR not only determines eligibility but may also predict clinical outcomes. Notably, patients with TNR values between 3 and 4 were reported to have the lowest 6- and 12-month survival rates, suggesting that a TNR > 4 may be associated with better tumor control, while a TNR < 3 may reflect slower-growing tumors with less metabolic activity [17]. Meningiomas, typically slow-growing and low-metabolism tumors, resemble the low-TNR group in this context. In our study, however, TNR did not significantly predict treatment response, which may be attributable to the limitations of Fluciclovine PET. This highlights the need for further research into more accurate surrogates for BPA uptake in meningioma.

Tumor location was another key factor affecting BNCT efficacy. Unlike conventional radiotherapy or particle therapy, which can deliver dose to deep-seated tumors, BNCT using epithermal neutrons has a limited penetration depth of approximately 8 cm. This limitation makes skull base meningiomas more difficult to treat effectively. Of the four patients with skull base meningiomas, three required dual-field irradiation to minimize dose to critical adjacent structures such as the brainstem. This approach was logistically challenging due to the lack of a robotic couch in the treatment room. Each change in patient position necessitated a reactor power-down period to allow radiation levels to decay before repositioning, significantly extending overall treatment time.

Previous BNCT studies have emphasized the importance of minimum tumor dose in achieving durable tumor control [39]. This is consistent with tumor control probability (TCP) models, which suggest that underdosed regions may compromise local control [40]. This concern is particularly relevant in BNCT, where central necrosis or hypoxic regions can impair BPA uptake. Additionally, BNCT’s dose distribution is highly localized at the cellular level, in contrast to the more diffused distribution of conventional radiotherapy. In our cohort, both mean and minimum tumor doses were higher in responders, but mean dose demonstrated a more robust association with treatment response. This may reflect the slow-growing nature of meningiomas, in which high-dose regions respond earlier while underdosed regions have yet to progress.

One limitation of our study is the heterogeneity of the patient cohort. While previous studies of BNCT in recurrent meningioma have focused primarily on higher-grade lesions [41], our study included only one patient with WHO grade 3 meningioma, alongside six patients with WHO grade 2 and six with WHO grade 1 meningioma. This distribution limits the generalizability of our findings to more aggressive meningioma subtypes. Further studies with a larger proportion of high-grade cases are needed to better evaluate the efficacy of BNCT in this subgroup.

Another limitation is the use of MRI-based RANO criteria to assess treatment response. Given the slow growth and regression characteristics of meningiomas, many patients were categorized as having stable disease. A longer follow-up period would allow for more accurate evaluation of treatment efficacy but requires additional time and resources. Alternatively, using the same PET tracer (e.g., Fluciclovine) in follow-up imaging may help assess metabolic activity and provide earlier insight into physiological treatment response. This approach will be explored in future studies.

## Conclusion

This study demonstrates that BNCT is a promising salvage treatment option for recurrent meningiomas, especially in non–skull base locations where favorable dose coverage can be achieved. Tumor mean dose was significantly associated with treatment response, underscoring the importance of precise dose planning. While Fluciclovine PET provides logistical advantages, its limitations in accurately reflecting BPA uptake highlight the need for improved imaging surrogates. Longer follow-up and metabolic imaging may enhance assessment of therapeutic efficacy in this patient population.

## Data Availability

No datasets were generated or analysed during the current study.
